# Human Milk Growth Factors and Their Role in NEC Prevention: A Narrative Review

**DOI:** 10.3390/nu13113751

**Published:** 2021-10-23

**Authors:** Daniel J. York, Anne L. Smazal, Daniel T. Robinson, Isabelle G. De Plaen

**Affiliations:** 1 Division of Neonatology, Department of Pediatrics, Ann & Robert H. Lurie Children’s Hospital of Chicago, Feinberg School of Medicine, Northwestern University, Chicago, IL 60611, USA; daniel.york@northwestern.edu (D.J.Y.); alsmazal@luriechildrens.org (A.L.S.); DTRobinson@luriechildrens.org (D.T.R.); 2 Center for Intestinal and Liver Inflammation Research, Stanley Manne Children’s Research Institute, Ann & Robert H. Lurie Children’s Hospital of Chicago, Northwestern University, Chicago, IL 60611, USA

**Keywords:** necrotizing enterocolitis, human milk, growth factors, Epidermal Growth Factor (EGF), Heparin-Binding EGF-like Growth Factor (HB-EGF), Insulin-like Growth Factor 1 (IGF-1) Insulin-like Growth Factor 2 (IGF-2), Vascular Endothelial Growth Factor (VEGF), Erythropoetin (EPO), Granulocyte Colony Stimulating Factor (G-CSF), Holder pasteurization

## Abstract

Growing evidence demonstrates human milk’s protective effect against necrotizing enterocolitis (NEC). Human milk derives these properties from biologically active compounds that influence intestinal growth, barrier function, microvascular development, and immunological maturation. Among these protective compounds are growth factors that are secreted into milk with relatively high concentrations during the early postnatal period, when newborns are most susceptible to NEC. This paper reviews the current knowledge on human milk growth factors and their mechanisms of action relevant to NEC prevention. It will also discuss the stability of these growth factors with human milk pasteurization and their potential for use as supplements to infant formulas with the goal of preventing NEC.

## 1. Introduction

Necrotizing enterocolitis (NEC) is the most frequently encountered disease affecting the premature infant intestine with an estimated incidence of 5 to 10% among very low birth weight (<1500 g) neonates [1]. NEC carries a mortality rate of about 20–30% [2]. Of babies who develop NEC, approximately 30% will require surgery. Additionally, long-term complications are associated with this disease including parenteral nutrition-associated cholestasis and liver dysfunction, poor growth/malnutrition, metabolic bone disease, short bowel syndrome, sepsis/severe infection, and neurocognitive impairment [3].

The incidence of NEC has been reported to be lower in premature infants fed human milk compared with those fed infant formula [4,5]. Human milk provides immunologic, nutritional and developmental benefits to the growing newborn via multiple molecular and cellular mechanisms. It is thought that growth factors (GFs) contained in human milk may mediate some of these mechanisms.

NEC is characterized by different degrees of intestinal necrosis likely resulting as the end point of several mechanistic pathways where factors such as abnormal bacterial colonization, immunologic immaturity, immature intestinal barrier function, and microvascular under-development play a role. This review discusses the current knowledge on the protective properties conferred by human milk GFs on the intestines after birth and when known, offers perspective on GFs present in the amniotic fluid prenatally. We review the mechanism of action (Table 1) and reported levels of GFs in breast milk (Table 2) and discuss the potential therapeutic applications for reducing NEC in high-risk neonates.

Question: In the clinical context of necrotizing enterocolitis, what are the developmental benefits elicited by human milk growth factors and how should these best be utilized from a therapeutic perspective?

Objectives:

1. List the growth factors contained within human milk that are shown to have clinical importance in preventing necrotizing enterocolitis.
2. For each growth factor, summarize levels in human milk and describe how levels change over time.
3. Describe each growth factor’s biochemical and cellular mechanism for augmenting intestinal health.
4. Summarize therapeutic trials of growth factors
5. Describe donor milk processing and effects on human milk growth factors.
6. Provide insight into the next steps required to establish therapeutic potential for each growth factor.

## 2. Methods

In a systematic search process, our team reviewed primary literature results written in English, from keyword queries in Ovid MEDLINE and Epub as well as Google Scholar. Texts with the following status: Ahead of Print, In-Process, In-Data-Review and Other Non-Indexed Citations, Daily and Versions from dates from 1946 to March 05, 2021 were assessed. From that search, 3683 articles matched search terms and 120 articles were included based on scientific rigor, impact and relevance to our topic.

Search Strategy:

1. exp Milk, Human/ or exp Infant Formula/ or (breast adj2 (milk or feed)).ti,ab,kw. or ((donor or mother*) adj3 milk).ti,ab,kw. (*n* = 31,935)
2. (intestin* adj5 (development or pathology)).ti,ab,kw. (*n* = 7381)
3. growth factor.ti,ab,kw. (*n* = 328,920)
4. exp “Intercellular Signaling Peptides and Proteins”/ (*n* = 1,078,972)
5. 3 or 4 (*n* = 1,216,365)
6. 1 and 5 (*n* = 1180)
7. exp Infant, Premature/ (*n* = 57,394)
8. exp Infant, Premature, Diseases/ or exp Infant, Low Birth Weight/ or exp Infant, Very Low Birth Weight/ (*n* = 73,576)
9. exp Infant, Premature/ or exp Infant, Premature, Diseases/ or exp Infant, Low Birth Weight/ or exp Infant, Very Low Birth Weight/ or (premature or preterm).ti,ab,kw. (*n* = 239,737)
10. 7 or 8 or 9 (*n* = 239,737)
11. 6 and 10 (*n* = 178)
12. limit 11 to english language (*n* = 174)
13. exp Enterocolitis, Necrotizing/ (*n* = 3683)
14. 1 and 5 and 13 (*n* = 67)
15. 12 or 14 (*n* = 193)
16. limit 15 to english language (*n* = 191)

## 3. Narrative

### 3.1. Epidermal Growth Factor (EGF)

Epidermal Growth Factor (EGF) is thought to decrease the susceptibility of infants to NEC via several mechanisms that are reviewed here. Data continue to emerge from both animal and human studies exploring the potential therapeutic use of EGF as an additive to pasteurized breast milk or to infant formulas [44,45]. Additionally, in utero, the fetal intestine is exposed to increasing levels of EGF in amniotic fluid as gestation progresses with the most rapid increase in concentration seen near term, a characteristic that suggests a role for EGF in the maturation of the intestines in late gestation [46,47].

EGF, a growth factor first discovered in saliva [48], exists at relatively high concentrations in human colostrum and decreases steadily in human milk throughout the first 2 months of lactation [9,38] to about half their initial levels in milk from women who delivered full term infants [38]. Concentrations of EGF in milk from women who delivered at 23–27 weeks of gestation were higher than in milk from mothers of term infants at similar stages of lactation [37]. Importantly, the Holder pasteurization method commonly used for donor breast milk processing does not reduce EGF concentrations [49].

EGF has been known since the early 1980s to play a role in intestinal epithelial growth, maturation, and development [50,51]. More specifically, EGF binds to EGF receptors (EGFR) and increases the proliferation rate of intestinal epithelial cells in adult mice within hours of exposure [6]. These early studies demonstrated increased intestinal size, weight, cell production, and digestive enzyme activity. In addition, EGF has recently been shown to promote the barrier function of intestinal epithelial cells [10]. In Caco-2 cells exposed to hydrogen peroxide, EGF prevents tight junction and actin cytoskeleton disruption, and reduces epithelial barrier permeability through MAPK (mitogen-activated protein kinase)-dependent mechanisms [8]. When EGF signaling is inhibited in dams, neonatal mice have increased bacterial translocation of pathogenic E. Coli in mesenteric lymph nodes, splenic tissue, or hepatic tissue samples. This further suggests a protective role for EGF/EGFR dependent signaling on intestinal barrier [9]. This translocation is thought to occur via goblet cell associated passages (GAP) which are suppressed by a functioning EGF pathway [9].

EGF also promotes intestinal epithelial repair and regeneration following injury [7]. In a neonatal rat model of NEC, EGF supplementation of rat milk substitute (RMS) decreased NEC incidence, decreased histologic NEC severity, reduced the ileal production of the pro-inflammatory cytokine IL18, and increased the production of interleukin (IL)-10 and of its transcription factor, Sp1, when compared with RMS alone [52]. More recently, EGF’s contribution to immunomodulation was further suggested by evidence that milk-derived EGF attenuates Toll-like Receptor4 (TLR4) signaling in the neonatal intestine [11], which is thought to contribute to the development of NEC [11,12].

In a step towards supplementing formula with EGF, genetically modified soybeans have been engineered to produce functional human EGF on an industrial scale [44]. In a rat model of NEC, soybean-derived EGF supplementation has been shown to improve intestinal barrier function, to reduce the expression of pro-inflammatory proteins Cyclooxygenase-2 (COX-2) and Inducible Nitric Oxide Synthase (iNOS), and to decrease the incidence of intestinal injury [44]. Given its protective effects in experimental models and its stability in the gastric environment [53], EGF wields potential as a preventative therapy among high-risk newborns. 

### 3.2. Heparin-Binding EGF-like Growth Factor (HB-EGF) 

Heparin-Binding EGF-like Growth Factor (HB-EGF) is a member of the EGFR family of ligands. First described in the 1990s as a product of human macrophages [54], HB-EGF is an autocrine signaling molecule secreted by many cell types, including macrophages, fibroblasts, smooth muscle cells and epithelial cells [39,55]. Its presence in both human milk and amniotic fluid suggests its importance in early development of the intestine.

While levels of HB-EGF are quantitatively less than those of EGF in human milk [39,55], HB-EGF has higher potency than EGF as it binds the EGF receptor with a much higher affinity than EGF. As opposed to EGF, previous studies did not show consistent differences in HB-EGF concentration between human milk from different post-conceptional age or post-natal age [39].

HB-EGF promotes epithelial cell proliferation, migration and wound healing [13], thus improving gut barrier function [17]. In addition to its ability to bind the EGFR, HB-EGF binds to N-arginine dibasic convertase to mediate cell migration via the EGF receptor ErbB1 [15]. Following ischemia/reperfusion injury, HB-EGF is upregulated and enhances intestinal epithelial cell restitution via Phosphoinositide 3-kinase (PI3K/Akt) and Mitogen-activated protein kinase/ Extracellular Signal-Regulated kinase (MEK/ERK1/2) [56]. Similar to EGF, HB-EGF also attenuates local inflammation [57] and intestinal epithelial cell apoptosis [22] in several models. Additionally, in rat NEC models, HB-EGF reduces production of reactive oxygen species (ROS) [18], promotes intestinal epithelial cell regeneration [14,16], attenuates apoptosis [22], and protects intestinal stem cells from injury [58].

In addition, HB-EGF appears to preserve intestinal microvillous blood flow in rats exposed to NEC stress [20]. HB-EGF induced angiogenesis in human umbilical vein endothelial cells [19] and enhanced vasodilation in ex vivo rat pup intestines [21]. While these studies support a protective effect of HB-EGF on the intestinal vasculature, more investigation is needed to better delineate the HB-EGF effect on the intestinal microcirculation in NEC. HB-EGF has not been trialed in humans.

### 3.3. Insulin-like Growth Factor (IGF-1 and IGF-2)

Insulin-like growth factors (IGF)-1 and IGF-2 are growth factors present in the serum mostly bound to IGFBPs which regulate their stability and bioavailability [59]. IGF-1 is produced by the placenta [60] and the liver [61]. It is a major regulator of fetal growth and development of most organ systems [62].

IGF-1 is present at high concentrations in the colostrum and its concentration in breast milk declines over the first 6 months of lactation [60,61,63,64]. Studies have reported either similar or higher concentrations of IGF-1 levels in preterm versus term human milk [40,41]. IGF-2 levels may increase during the first week of lactation [65], and subsequently slowly decline over several months. No significant differences in IGF-2 levels were noted between term and preterm human milk. [41]

When bound to IGF Binding Protein 2 (IGFBP-2), IGF-1 and IGF-2 are largely stable when isolated and incubated in gastric fluid obtained from human neonates [41]. Without this chaperone binding interaction, IGF-1, IGF-2, and IGFBP are rapidly cleaved by gastric enzymes [41]. IGF-1 and IGF-2 were significantly reduced following Holder pasteurization by 39.4% and 9.9%, respectively [66].

IGF-1 promotes cell survival similarly to HB-EGF, primarily by inhibiting cell apoptosis through the PI3K/Akt signaling pathway [28]. In the intestine, IGF-1 has been shown to stimulate proliferation of intestinal stem cells [67] and to promote the survival of crypt cells following murine models of radiation injury [68,69] and oxidative injury [70]. IGF-1 administered enterally protects against NEC in rats by protecting the intestinal mucosal barrier [24] and by reducing the inflammatory response [24]. In the same model, enterally administered IGF-1 suppresses TLR4, nuclear factor kappa-light-chain-enhancer of activated B cells (NF-κB), and Interleukin (IL)-6 mRNA levels [24]. Systemic administration of IGF-1-BP3 complex protects against NEC in pigs [71] and in neonatal mice (unpublished data). Additionally, IGF-1, when administered intraperitoneally in conjunction with erythropoietin, protects the murine intestine against injury and cellular apoptosis in a model of hypoxia/reperfusion [27]. IGF was also shown to enhance intestinal absorption of nutrients such as D-glucose, L-alanine, and ions in a healthy piglets [23]. The summation of these effects is reduced apoptosis and an attenuated inflammatory response in models of intestinal tissue injury.

In the mouse neonatal intestine, IGF-1 secreted by resident macrophages promotes endothelial cell proliferation and microvascular development and protects against NEC (our lab—unpublished data). Further, systemic exogenous administration of IGF-1 to neonatal mice protects against NEC and maintains mucosal microvasculature integrity (our lab—unpublished data). Both IGF-1 and IGF-2 have been shown to independently promote cell migration of human umbilical cord vascular endothelial cells (HUVEC) in vitro and formation of their tubular structures (primitive vessels) [25,26]. In summary, growing evidence derived from animal studies indicates that the IGFs protect the integrity of the developing intestinal epithelium through proliferative, antiapoptotic and proangiogenic influences.

In a randomized control trial involving 60 very low birth weight (range: 750 to 1250g) neonates, IGF-1, when added to formula at twice the dose present in colostrum, showed improved gut barrier function at day 14 as evidenced by lower lactulose/mannitol excretion ratios in babies receiving IGF-1. [72]. However, this effect was not sustained when evaluated at later post-natal days. Additionally, no effect of enterally administered IGF-1 was noted on feeding tolerance nor weight gain [72]. Intravenous administration of human recombinant IGF-1 appears to be well tolerated in short term follow up in phase-II clinical trials [73,74]. Larger studies are needed to determine whether enterally administered IGF-1 may protect against NEC in humans.

### 3.4. Vascular Endothelial Growth Factor (VEGF)

Vascular Endothelial Growth Factor is a growth factor present in breast milk at much higher concentrations than found in human serum [42]. It is a member of a superfamily of related growth factors that include VEGF-A, B, C, D, E and placental derived growth factor (PlGF). VEGF-A, commonly referred to as VEGF, plays the most prominent role in regulating vascular angiogenesis during homeostasis and disease [75,76].

VEGF is present at relatively high concentrations in the human milk [40,77,78,79]. In a cohort of 43 mother-baby dyads, mothers who delivered infants at term demonstrated VEGF concentrations (>75 ng/mL) that were greater than milk from mothers who delivered before 37 weeks (30–40 ng/mL). Yet, findings are inconsistent between studies as to whether VEGF is higher in term or preterm milk [78,79]. In a cohort of term infants, VEGF concentrations in human milk were found to decrease as lactation progressed [77]

Mechanistically, cellular hypoxia allows for stabilization of hypoxia inducible factor (HIF) which in turn serves as a transcription factor for upregulation of VEGF [80]. The VEGF family of proteins binds to tyrosine kinase receptors located predominantly on the surface of vascular endothelial cells [81]. Upon binding to VEGFR2, VEGF induces intracellular signal transduction via the *notch* pathway and activation of the phosphatidylinositol 3-kinase/Akt pathway, to promote endothelial cell proliferation, migration and survival [29].

VEGF promotes vascular development (angiogenesis) in most organs. In very low birth weight infants, VEGF dysregulation may be associated with impaired microvascular development leading to organ dysfunction and increased morbidity. VEGF has a well demonstrated role in the pathogenesis of ROP, but there is mounting literature showing that it may contribute to pulmonary hypertension commonly associated with bronchopulmonary dysplasia [82]. Similarly, there is increasing evidence that defective VEGFR2 signaling may also play a role in NEC [83,84] and restoring VEGF production has been shown to preserve intestinal endothelial cell proliferation and to decrease the incidence of severe NEC in neonatal mice [85]. Further examination is needed to determine whether human milk-derived VEGF protects the neonatal intestines from NEC. In summary, VEGF dysfunction may contribute to several complications of prematurity.3.5. Erythropoetin (EPO):

Erythropoetin (EPO) is a glycoprotein produced in the liver and the kidneys that stimulates erythropoiesis primarily in response to cellular hypoxia. Functional EPO receptors are present on fetal and neonatal intestinal cells and regulate EPO’s function [86].

Both human milk and amniotic fluid contain EPO [87]. Amniotic fluid EPO levels increase in response to fetal hypoxia, as seen with exposure to maternal hypertension or preeclampsia, suggesting that EPO from swallowed amniotic fluid may offer fetal intestinal protection from these hypoxic conditions in utero [88]. In Human milk, EPO levels increase as lactation duration increases [89]. EPO is present in similar levels in both term and preterm human milk [43] (Pre/Term: 11.7 mU/mL) as measured in the first 4 months of life.

Much like EGF, HB-EGF and IGF-1, EPO promotes intestinal villous integrity via stimulation of intestinal epithelial cellular proliferation and migration. In the postnatal period, suckling rats whose mothers were supplemented with recombinant EPO showed increasing villous number and small bowel length [30]. A lesser but significant effect was seen for neonatal rats who received parenteral EPO [90]. This effect was mediated in part by EPO-induced migration of intestinal epithelial cells and resistance to Tumor Necrosis Factor (TNF)-induced apoptosis. In experimental NEC, EPO protects the intestinal epithelium by diminishing excessive autophagy via the Akt/ mechanistic target of rapamycin (mTOR) signaling pathway and by upregulating B-Cell Lymphoma gene 2 (Bcl-2) via the MAPK/ERK pathway to reduce apoptosis [90].

EPO contributes to intestinal barrier integrity by preserving tight junctions. Specifically, EPO increases the expression of zona occludens-1 (ZO-1) via the PI3K/Akt pathway. ZO-1 binds multiple tight junction-associated proteins and the peri-junctional actin ring and is vulnerable to proinflammatory cytokines including interferon-gamma (IFN-γ) [31]. In vitro, EPO was shown to reverse IFN-γ-induced downregulation of ZO-1 and alteration of the intestinal barrier [31]. Additionally, EPO attenuated the secretion of stimulated IL-8 from intestinal epithelial cells stimulated with TNF-α and IL-1β in vitro [32]. In vivo, oral administration of EPO decreased the incidence of experimental NEC in neonatal rats and prevented the loss of ZO-1 at tight junctions [31]. Thus, EPO appears to attenuate the inflammatory cascade, promote healthy villous epithelial regeneration, and optimize intestinal epithelial barrier function. Investigations of whether EPO prevents or mitigates NEC in preterm infants reveal mixed results. In a randomized control trial of infants <32 weeks gestational age, repeated dosing of intramuscular EPO decreased the incidence of Bell stage II and III NEC by 36 weeks postmenstrual age [91]. The trial’s limitations included lack of blinding to the study groups and a high reported incidence of NEC in the study cohort (17.1%), although notably, this included babies with Bell Stage I NEC, but is substantially higher than the incidence seen in the Vermont-Oxford Network of NICUs (7.6%) [92]. In a multicenter, randomized, double blind trial, intravenous EPO had no impact on the incidence of NEC in infants born at less than 28 weeks [93]. Another smaller trial administered enteral EPO in an artificial amniotic fluid solution to infants born at less than 28 weeks gestational age. Here, EPO had no effect on the incidence of NEC [94]. In a small randomized control trial, infants given enteral EPO demonstrated earlier tolerance of full feeding volumes with improved weight gain [95]. While EPO appears to be well tolerated when administered to human neonates, large-size trials are needed to conclusively determine whether exogenous EPO confers a preventative or therapeutic effect against NEC.

### 3.5. Granulocyte Colony Stimulating Growth Factor (G-CSF)

Granulocyte colony stimulating factor (G-CSF) is produced by the developing fetus and placenta both for maintenance of hematopoiesis and in response to inflammation [96,97,98]. As a GF, G-CSF elicits wide ranging effects apart from those for which it was named (leukopoiesis). Its therapeutic role has been evaluated in both humans and animal models and its clinical use among neonates remains to be established.

G-CSF is present in relatively high amounts in amniotic fluid and human colostrum, though it is notably decreased in preterm milk [96]. Measured on day of life 2, term levels are reported in the range of 0.0156 µg/100mL, while preterm are lower at 0.0080 µg/100mL. No data are available on the levels of G-CSF in milk produced at advanced lactational stages.

G-CSF is a glycoprotein that promotes proliferation and differentiation of granulocytes and neutrophils via the Janus Tyrosine Kinase/ signal transducers and activators of transcription (Jak/STAT) and Mek/Erk pathways but also has a variety of nonhematopoietic effects much like EPO [99]. Enteral G-CSF is not systemically absorbed, but rather binds to enteral villus receptors and stimulates growth and development of the fetal bowel [35,36,100]. Interestingly, both human and recombinant G-CSF resist gastric degradation in human milk, but are degraded when added to infant formula [101].

G-CSF promotes gut barrier integrity and epithelial cell health in the neonatal intestine. In a rat model of hypoxia-reoxygenation, enterally administered G-CSF reduced histopathologic evidence of mucosal damage in the small and large intestines [33]. In rodent models of chemotherapy-induced intestinal injury and permeability, G-CSF partially prevented bacterial translocation by reducing apoptosis and restoring villus height [102]. In contrast, in a mouse model of NEC, subcutaneous G-CSF during NEC induction increased the severity of intestinal inflammation and tissue damage [103]. This effect was dependent on neutrophil activity, as transgenic mice lacking neutrophil elastase, a serine protease essential for neutrophil activity, did not develop NEC even if given subcutaneous G-CSF [103].

G-CSF has been studied as a potential therapy for infants with neutropenia and sepsis. Neutropenia may be a risk factor for the development of NEC and has been associated with worsened prognosis [104,105]. Using G-CSF to prevent or improve neutropenia could in theory offer some protection from the development of NEC. Administration of G-CSF improves recovery of absolute neutrophil count while decreasing all-cause mortality in preterm infants with sepsis and neutropenia [106,107]. Beyond augmenting neutrophil counts, recombinant G-CSF may improve neutrophil function by increasing phagocytosis and oxidative burst activity [106]. However, in a large cohort of neutropenic infants, exogenous G-CSF was associated with increased incidence of secondary sepsis despite improvement in neutropenia [108]. In a multicenter, randomized controlled trial, prophylactic treatment of neutropenic premature infants was not associated with significant differences in survival free of infection [109]. Given these conflicting results, G-CSF is not routinely given to neutropenic infants, and its role in preventing NEC requires further investigation. In a small pilot study of Bell stage 1 (suspected) NEC, infants who received enteral G-CSF for 5 days in addition to standard treatment of withheld enteral nutrition, antibiotics, and gastric decompression had reduced incidence of progression to stage II or III NEC and faster clinical and radiologic resolution resulting in reduced duration of systemic therapy and reduced length of hospital stay [110]. In another randomized control trial, enteral G-CSF administered to preterm infants was, similarly to EPO, associated with improved feeding tolerance and decreased incidence of NEC despite no increase in serum G-CSF levels [95]. While these studies suggest that G-CSF could have therapeutic potential when administered enterally, results need to be replicated in additional randomized trials and further study is required to elucidate its mechanism of action in human neonates.

### 3.6. Donor Milk and Holder Pasteurization’s Effects on Growth Hormone Levels in Human Milk

Pasteurized human donor milk is recommended for feeding preterm infants when maternal breast milk is unavailable [111]. Donor milk is the product of pooled human milk from mothers at various stages of lactation, having delivered at wide ranges of pregnancy duration. Holder pasteurization, one of the most common methods used for pasteurization by donor milk banks, heats the human milk to 62.5 degrees Celsius for 30 min. The objective of the pasteurization method is to reduce the potential for transmission of infectious particles via the donor milk [49,66]. Yet, this method reduces concentrations of some human milk components known to be beneficial to the preterm neonate.

As an unintended consequence of heating, growth factor concentrations may be reduced via protein denaturation and degradation and the pasteurization method variably affects concentrations of different growth factors [112,113,114]. Of the growth factors reviewed in this article, EGF and HB-EGF concentrations do not significantly change with pasteurization. In contrast, IGF-1, IGF-2, and EPO were all reduced by pasteurization, and insufficient data are available for G-CSF [49,66,115]. It is possible that lower temperature or reduced exposure time to heat may help preserve growth factor biologic activity while providing adequate protection against infection [116].

Donated milk that is pooled and processed for infant feedings is often milk expressed at later stages of lactation. As established in this review, many GF vital to the premature neonate’s intestinal development decrease in concentration as lactation progresses. [112,113]. Therefore, the conditions that ultimately result in batches of donor milk utilized by NICUs likely lead to providing preterm infants milk that has relatively lower concentrations of GF [113]. Further studies are required to examine the net effect of donor milk processing on GFs.

Donor milk reduces risk of NEC [117,118,119,120]. In a retrospective cohort analysis of 319 neonates of very low birth weight (VLBWs), infants received either their mother’s own milk and donor human milk, or their mother’s own milk and formula. Feedings consisting in mother’s own milk and donor milk were associated with a significant reduction of the incidence of NEC when compared with the group receiving their mother’s own milk and formula (1.8% vs. 6.0%, *p* = 0.048). Further, a meta-analysis of five randomized control trials showed that donor milk when compared with preterm formula reduces the risk of NEC by up to 79% in the combined analysis. This suggests that the protective effects remain despite the reduced concentrations of growth factors resulting from pasteurization, and/or other components preserved in pasteurization offset the reduction in concentrations of protective growth factors [121].

## 4. Limitations

This narrative review is meant to provide an overview to the reader of both what is known and knowledge gaps requiring further study. It attempts to summarize decades of growth factor research and put it in the context of necrotizing enterocolitis. We do not provide additional data nor attempt to reanalyze the data presented. Despite critical and rigorous evaluation of each study included here, narrative reviews are generally subjected to authors’ experiential biases.

## 5. Conclusions

**Figure 1:**
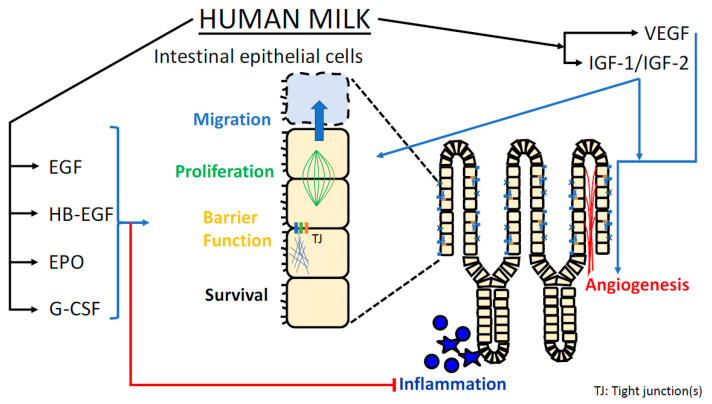
Graphical representation of mechanisms by which growth factors present in breast milk may protect the neonatal intestine against NEC.

NEC carries severe risks including sepsis, need for bowel resection, intestinal failure, and death. Even milder cases compromise infant growth, increase antibiotic exposure, delay time to full enteral feeds, increase cost burden, and prolong length of stay in the NICU. Human milk feedings, particularly their mother’s own milk, remain a potent preventative strategy against the development of NEC. However, even infants fed exclusively their mother’s own milk may develop NEC. Our extensive literature search draws important similarities among the effects imparted by various growth factors contained within breast milk. Common mechanisms include augmented intestinal restitution, prolonged intestinal stem cell survival, improved intestinal perfusion, and enhanced intestinal cellular repair and migration (Figure 1).

Understanding the components of human milk that confer protection and the influences of their concentrations in milk may offer opportunities for the augmentation of human milk’s preventative and therapeutic effects in infants at risk of NEC. This may include the addition of growth factors to the milk of both term and preterm neonates. It is important to note that milk concentration does not necessarily imply bioactivity and bioactivity is not necessarily a linear correlation with concentration. Thus, any preclinical or phase I trials examining the effects of supplementary GFs, must evaluate a dose response curve. Alternative methods for the pasteurization of donor milk should be explored with the goal of preserving all immunomodulators within breast milk. Increased understanding of how human milk growth factors function in concert with other nutrients in milk may best reveal opportunities to enhance the protective effects of these human milk proteins.

With the establishment of large-scale production of human growth factors (such as that achieved with EGF), the possibility of growth factor supplementation in breast milk is materializing. From a translational perspective, safety data from animal models must continue to demonstrate that GFs produced from other organisms (soybeans) is biologically safe and stable. Ensuing phase I clinical trials would likely need to establish safety in healthy neonates prior to the introduction to fragile preterm infants. While GF supplementation may help intestinal development, if supplemented at levels seen in breast milk, at best, GFs should be expected to decrease NEC only to the levels seen in babies exclusively fed breast milk. Future studies should continue to explore the mechanisms of intestinal development and the pathophysiology of NEC to elucidate early biomarkers of the disease.

## Figures and Tables

**Table 1:** A summary of mechanisms by which each growth factor offers protection from NEC in the neonatal intestines. Additionally, information regarding stability of the compound orally (gastric secretions) and following Holder pasteurization is shown.

Hormone	Role in NEC Protection	Stable	
		Orally	Holder Pasteurization
EGF	Increases proliferation of intestinal epithelium [6] Enhances appropriate cellular migration in intestinal healing [7] Optimizes epithelial barrier function [8,9,10] Attenuates TLR4-mediated inflammatory response [11,12]	Yes	Yes
HB-EGF	Stimulates intestinal cell proliferation [13,14,15,16] Promotes epithelial barrier function [17] Reduces production of ROS [18] Augments microvascular perfusion (angiogenesis, vasodilation) [19,20,21] Attenuates apoptosis [22]	Yes	Yes
IGF-1/ IGF-2	Promotes epithelial barrier function [23,24] Reduces inflammatory response [24] Promotes mucosal microvascular development [25,26] Attenuates apoptosis [27,28]	Yes	Partially
VEGF	Promotes mucosal microvascular development [29]	Not reported	
EPO	Trophic effects on intestinal villi [30] Promotes epithelial barrier function [31] Reduces epithelial cell inflammatory response [32]	Yes	Partially
G-CSF	Accelerates mucosal healing [33] Trophic effect on intestinal villi [34,35,36]	Yes	Partially

EGF = epidermal growth factor, TLR4 = Toll-like Receptor 4, HB-EGF = Heparin binding EGF-like growth factor, ROS = Reactive Oxygen Species, IGF-1 = insulin like growth factor 1, IGF-2 = insulin like growth factor 2, VEGF = vascular endothelial growth factor, EPO = erythropoietin, G-CSF = granulocyte colony-stimulating factor.

**Table 2:** Human milk growth factor concentrations with comparisons by preterm birth status and stage of lactation.

Growth Factor	Preterm vs. Term Milk	Statistical Differences between Groups	Early vs. Late Lactation	Statistical Differences between Stages of Lactation
EGF	Prem: 16–17 µg/100 mL Pre/Term: 10–11 µg/100 mL [37]	Yes	DOL 3-5: ~15 µg/100 mL DOL 31-60: ~8 µg/100 mL [38]	Yes (among both term and preterm populations)
HBEGF	Range: 2×10^−3^ –2.3×10^−2^ g/100 mL [39] Gestational age not reported	Not reported	No clear trend	No
IGF1	Term: 0.031 µg/100 mL [40] Prem: 0.419 µg/100 mL [40] At DOL 28	Yes	Preterm milk significant increase in IGF-1 0.231–0.419 µg/100 mL over DOL 3-28 [40]	Yes
IGF2	Term: 1.2 µg/100 mL [41] Prem: 1.22 µg/100 mL [41]	No	Slight decline over first 3 months [41]	No
VEGF	Term: ~8 µg/100 mL Prem: ~3 µg/100 mL [42] Measured within first 7 DOL	Yes	Decline in Term levels from 8 to ~2.5 µg/100 mL. Not reported for premature infants.	Yes
EPO	Pre/Term: 11.7 mU/mL [43] Measured in first 4 months of life	No	Increases with duration of lactation, highest mean level 33.8 mU/mL measured over DOL 51–134 [43]	Yes
G-CSF	Term: 0.0156 µg/100 mL Prem: 0.0080 µg/100 mL [34] Measured on DOL 0–2	Yes	Not reported	Not reported

Prem = Premature birth, DOL = Days of Life of baby, Pre/Term = Preterm + Term gestation. EGF = epidermal growth factor, HB-EGF = Heparin binding EGF-like growth factor, IGF-1 = insulin like growth factor 1, IGF-2 = insulin like growth factor 2, VEGF = vascular endothelial growth factor, EPO = erythropoietin, G-CSF = granulocyte colony-stimulating factor, mU = milliunits.

## Data Availability

Not Applicable.

## Abbreviations

EGF: epidermal growth factor; EGFR: epidermal growth factor receptor; EPO: erythropoietin; G-CSF: granulocyte colony-stimulating factor; GAP: goblet cell associated passages; GF: growth factor; HB-EGF: heparin-binding epidermal growth factor; HIF: hypoxia inducible factor; HUVEC: human umbilical cord vascular endothelial cells; IFN-γ: interferon gamma; IGF-1 and 2: insulin-like growth factor 1 and 2; IGFBP: Insulin-like growth factor binding protein; IL: interleukin; MAPK: mitogen-activated protein kinase; NEC: necrotizing enterocolitis; PIGF: placental derived growth factor; ROP: retinopathy of prematurity; ROS: reactive oxygen species, TLR: Toll-like receptor, TNF: tumor necrosis factor; VEGF: vascular endothelial growth factor; VEGFR: vascular endothelial growth factor receptor; VLBW: very low birth weight; ZO-1: zona occludens-1, PI3K/Akt: Phosphoinositide 3-kinase, MEK/ERK1/2: Mitogen-activated protein kinase/ Extracellular Signal-Regulated kinase, COX2: Cyclooxygenase-2, iNOS: Inducible Nitric Oxide Synthase, NF-κB: nuclear factor kappa-light-chain-enhancer of activated B cells, mTOR: mechanistic Target of Rapamycin, Bcl2: B-cell Lymphoma gene 2 JAK/STAT: Janus Tyrosine Kinase/ signal transducers and activators of transcription
